# De Novo Transcriptome Sequencing of *Codonopsis lanceolata* for Identification of Triterpene Synthase and Triterpene Acetyltransferase

**DOI:** 10.3390/ijms24065769

**Published:** 2023-03-17

**Authors:** Han-Bin Choi, Sangrea Shim, Myeong-Hyeon Wang, Yong-Eui Choi

**Affiliations:** 1 Department of Bio-Health Convergence, Kangwon National University, Chuncheon 24341, Republic of Korea; 2 Department of Forest Resources, College of Forest and Environmental Sciences, Kangwon National University, Chuncheon 24341, Republic of Korea

**Keywords:** transcriptome analysis, triterpene, 2,3-Oxidosqualene cyclase, Codonopsis lanceolata

## Abstract

*Codonopsis lanceolata* (Campanulaceae) is a perennial plant commonly known as the bonnet bellflower. This species is widely used in traditional medicine and is considered to have multiple medicinal properties. In this study, we found that shoots and roots of *C. lanceolata* contained various types of free triterpenes (taraxerol, β-amyrin, α-amyrin, and friedelin) and triterpene acetates (taraxerol acetate, β-amyrin acetate, and α-amyrin acetate). The content of triterpenes and triterpene acetates by GC analysis was higher in the shoot than in the roots. To investigate the transcriptional activity of genes involved in triterpenes and triterpene acetate biosynthesis, we performed de novo transcriptome analysis of shoots and roots of *C. lanceolata* by sequencing using the Illumina platform. A total of 39,523 representative transcripts were obtained. After functional annotation of the transcripts, the differential expression of genes involved in triterpene biosynthetic pathways was investigated. Generally, the transcriptional activity of unigenes in the upstream region (MVA and MEP pathway) of triterpene biosynthetic pathways was higher in shoots than in roots. Various triterpene synthases (2,3-oxidosqualene cyclase, OSC) participate to produce triterpene skeletons by the cyclization of 2,3-oxidosqualene. A total of fifteen contigs were obtained in annotated OSCs in the representative transcripts. Functional characterization of four OSC sequences by heterologous expression in yeast revealed that ClOSC1 was determined as taraxerol synthase, and ClOSC2 was a mixed-amyrin synthase producing α-amyrin and β-amyrin. Five putative contigs of triterpene acetyltransferases showed high homology to the lettuce triterpene acetyltransferases. Conclusively, this study provides the basis of molecular information, particularly for the biosynthesis of triterpenes and triterpene acetates in *C. lanceolata*.

## 1. Introduction

*Codonopsis* is a genus containing 42 species of herbaceous perennial plants in the family Campanulaceae, predominantly found in Central, East, and South Asia. Several species of *Codonopsis* are widely used in traditional medicine with a long history around the world and are considered to have various pharmacological activities [1,2]. The roots of some species are used as foods in Asia [1]. Almost no obvious toxicity or side effect is observed and recorded for *Codonopsis*. Triterpenoid saponins are the main compounds from the *Codonopsis* species [1]. In addition to saponins, various types of sterol, triterpenes, and triterpene esters are found in the genus *Codonopsis* [1].

*C. lanceolata* is mainly distributed in East Asia, including Korea, Japan, China, and the Russian far east, and has been used for the treatment of respiratory diseases such as bronchitis, coughs, asthma, and tuberculosis [3]. Although several triterpenes and triterpene glycosides (saponins) were reported in some articles [4,5,6], there have been no reports about the simultaneous identification of various triterpenes in the roots and leaves of *C. lanceolata*. 

Triterpenes in plants constitute a large and structurally diverse group of natural products with various functions [7]. Triterpenes exhibit various biological and pharmacological activities [8,9]. Triterpenes are synthesized mainly via the mevalonate (MVA) pathway [10,11]. The cyclization of 2,3-oxidosqualene is the first step to form diverse triterpene skeletons catalyzed by oxidosqualene cyclases (OSCs). In plants, various OSCs participated to produce triterpene skeletons by the cyclization of 2,3-oxidosqualene [7]. These enzymes exist as supergene families in the plant genome. The genes involved in triterpenes and saponin biosynthesis in *C. lanceolata* have not been identified.

Acylated triterpenes esterified by acetic acid and fatty acids are very common in many plant species [12,13]. Despite the widespread occurrence of triterpene esters in plants, there is little or no information on the genes participating in the production of triterpene esters from free triterpenes. Recently, the lettuce LsTAT1 enzyme is functionally characterized as a pentacyclic triterpene acetyltransferase, particularly for the production of taraxasterol acetates and ψ-taraxasterol acetates [14].

Transcriptome sequencing analysis studies provide a powerful method to discover novel genes involved in target metabolite biosynthesis. Next-generation sequencing (NGS) has been used in various medicinal plant species [14,15,16]. A transcriptomic study was reported only for *Codonopsis pilosula* for the identification of genes involved in polysaccharide biosynthesis [17,18]. In a relative species, *Platycodon grandiflorum,* which is belonging to the family Campanulaceae, several transcriptomic approaches have been done for the identification of candidate genes involved in triterpene biosynthesis [19,20,21,22]. Despite the commercial and medicinal importance of *C. lanceolata*, a transcriptomic approach has not yet been performed.

In the current study, we identify the various triterpenes and triterpene acetates in the roots and shoots of *C. lanceolata*. De novo transcriptome sequencing analysis of *C. lanceolata* was conducted using the Illumina platform. We analyzed the transcriptional activity of genes involved in triterpene biosynthesis, and functionally characterized the triterpene synthase genes involved in triterpene production in *C. lanceolata*.

## 2. Results

### 2.1. Accumulation of Triterpenoids in Different Organs of C. lanceolata

**Figure 1:**
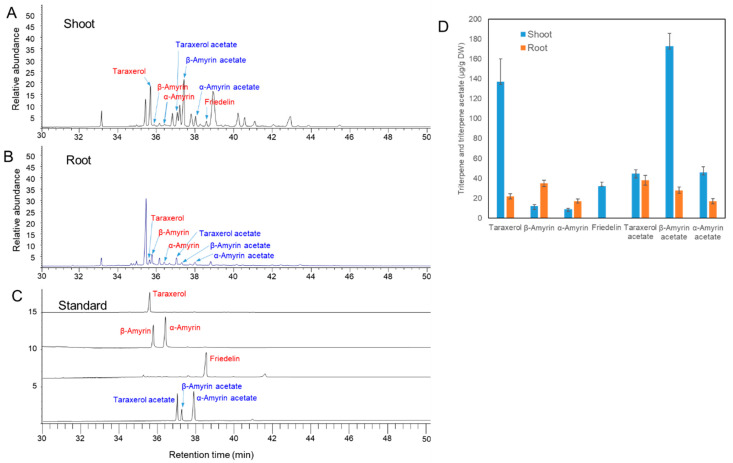
Identification of free triterpenes and triterpene esters by GC/MS analysis of *C. lanceolata* shoot and root extracts. (**A**) Total ion chromatogram (TIC) of shoot extracts. (**B**) TIC of root extracts. (**C**) TIC of authentic triterpene and triterpene acetate standards. (**D**) Content of triterpenes and triterpene acetates in shoots and roots.

Although a large number of triterpenes have been identified in the genus *Codonopsis* [1], only a few triterpenes such as α-spinasterol [23], friedelin [24], oleanolic acid [25], and echinocystic acid [25] are identified in *C. lanceolata* plants. In this work, we identified the four triterpenoids and three triterpene esters by GC-MS in the root and shoots of *C. lanceolata*, based on a comparison of their retention times and MS fragmentation patterns with those of standard compounds. GC/MS analysis of the shoots and the roots of two-month-old *C. lanceolata* plants revealed that four triterpenes (taraxerol, β-amyrin, α-amyrin, and friedelin) and three triterpene acetates (taraxerol acetate, β-amyrin acetate, and α-amyrin acetate) were presented in shoots and/or roots (Figure 1A,B), which were characterized by retention times and mass fractions of standard compounds (Figure 1C). These triterpenes and triterpene acetates, except the β-amyrin and α-amyrin, were presented in a richer amount in shoots than in roots (Figure 1D). 

### 2.2. Transcriptome Analysis of C. lanceolata and Functional Annotation of Unigenes

**Figure 2:**
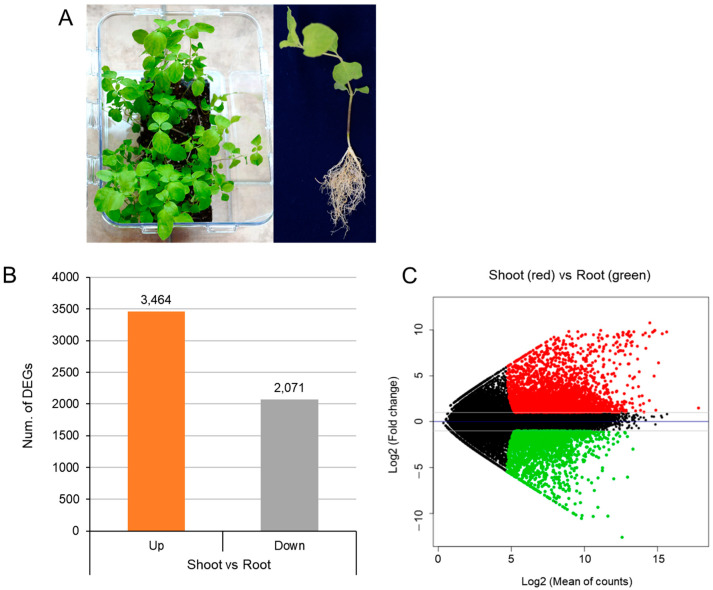
Differentially expressed genes (DEGs) expressed from transcriptome data of *C. lanceolata* shoots and roots. (**A**) Photo of two-month-old *C. lanceolata* plants for sequencing. (**B**) Number of DEGs highly expressed in both shoots and roots of *C. lanceolata*. (**C**) MA-plot for differential expression analysis in shoots and roots RNA-seq samples with three repeated samples, annotated to illustrate the use of the grammar of graphics. Points is our geometric object, x axis indicates the normalized mean and the y axis indicates the log2 fold change. Red and green dots represent the upregulated and downregulated DEGs, respectively.

To obtain high-quality unigenes by transcriptome analysis in the samples of shoots and roots of two-month-old plants (Figure 2A) using next-generation sequencing (NGS) technology, a total of six mRNA samples from two different organs (root and shoot) (n = 3 for each) were sequenced using the Illumina HiSeq X Ten System (Illumina, San Diego, CA, USA). A total of 130,340,370 reads (19,681,395,870 bp) were generated. After trimming the adapter and low-quality reads and removing those shorter than 25 bp, a total of 125,053,390 high-quality reads were obtained from the combined six samples. These reads were assembled into a total of 101,985 contigs (or transcripts) (N50 = 1993 bp), from which 39,523 representative transcripts were obtained. 

Annotation analysis was performed using 39,523 representative unigenes using two public databases, such as NCBI nonredundant protein sequences (NR) and InterPro database (http://www.ebi.ac.uk/InterPro/ (accessed on 15 January 2022)). The transcript abundance of each unigene was calculated through the Relative Log Expression (RLE) normalization method. After the analysis, 21,580 (54.6%) transcripts were annotated based on the information in all these databases. Finally, a sum of 5535 DEGs was identified in which 3464 unigenes were up-regulated in the shoot while 2071 unigenes were downregulated (Figure 2B). The MA-plot shows the log average for differential expression analysis in shoots and roots RNA-seq samples (Figure 2C).

**Figure 3:**
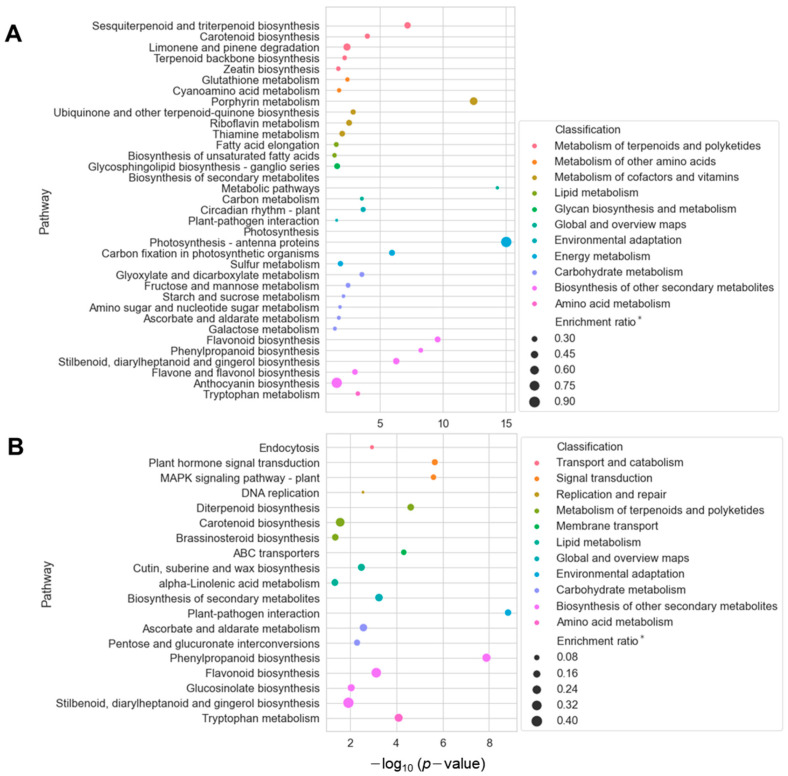
KEGG pathway enrichment analysis for up-regulated DEGs in shoot and root samples. A-B, Enriched pathways for a total of 3464 and 2071 DEGs upregulated in shoots (**A**) and roots (**B**) are shown, respectively (*p*-value < 0.05). The X and Y axes represent the statistical significance and name of the overrepresented pathways, respectively. The color of the dots indicates a higher-order classification of the enriched pathway. Enrichment ratio * (size of dots) represents the ratio of the number of DEGs to the total number of reference genes in a particular pathway.

To gain an insight into the molecular functions of selected DEGs, we conducted a functional enrichment analysis based on the annotations in the Kyoto Encyclopedia of Genes and Genomes (KEGG) database. Intriguingly, we found that several molecular pathways belonging to terpenoid and polyketide metabolisms were enriched for both upregulated DEGs in shoot and root samples (Figure 3). Consistent with the accumulation of triterpenoids and triterpene esters in GC-MS analysis, sesquiterpenoid/triterpenoid and diterpenoid biosynthesis pathways were upregulated in the shoots and roots, respectively (Figure 3A,B).

### 2.3. Transcriptional Activity of Unigenes Involved in Triterpene Biosynthetic Pathways 

**Figure 4:**
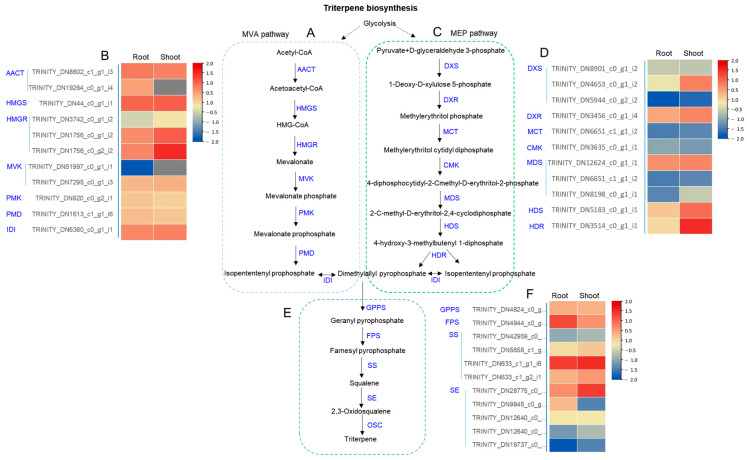
Biosynthetic pathway of triterpenes via MVA and MEP pathways and heat map of genes expressed in shoots and roots of *C. lanceolata* transcripts. (**A**) The MVA pathway produces two five-carbon building blocks called isopentenyl pyrophosphate (IPP) and dimethylallyl pyrophosphate (DMAPP), which are used to make isoprenoids. AACT: acetyl-CoA C-acetyltransferase; HMGS: 3-hydroxy-3-methylglutaryl-CoA synthase; HMG-CoA: 3-hydroxy-3-methylglutaryl-CoA; HMGR: 3-hydroxy-3-methylglutaryl-CoA reductase; MVK: mevalonate kinase; PMK: phosphomevalonate kinase; PMD: diphosphomevalonate decarboxylase; IDI: isopentenyl-diphosphate delta-isomerase. The transformation between Isopententenyl pyrophosphate and dimethylallyl pyrophosphate proceeds through IDI, which is a reversible reaction. (**B**) Heat map of annotated transcripts in MVA pathway expressed in shoots and roots of *C. lanceolata.* The color from red to blue indicates high to low expression levels. (**C**) MEP pathway is an alternative metabolic pathway for the biosynthesis of the IPP and (DMAPP), occurs in chloroplasts. Enzyme involved in the MEP pathway. DXS, deoxyxylulose 5-phosphate synthase; DXR, deoxyxylulose 5-phosphate reductoisomerase; MCT, 2-C-methyl-D-erythritol 4-phosphate cytidylyltransferase; CMK, 4- (cytidine 50 -diphospho)-2-C-methyl-D-erythritol kinase; MDS, 2-C-methyl-D-erythritol 2,4- cyclodiphosphate synthase; HDS, 4-hydroxy-3-methylbut-2-enyl diphosphate synthase; HDR, 4-hydroxy-3-methylbut-2-enyl diphosphate reductase (**D**) Heat map of annotated transcripts in MEP pathway expressed in shoots and roots. The color from red to blue indicates high to low expression levels. (**E**) Two molecules of IPP and one molecule of DMAPP are condensed to generate geranyl pyrophosphate and finally produce triterpenes. Enzyme involved in triterpene biosynthesis. GPPS, geranyl diphosphate synthase; FPS, farnesyl diphosphate synthase; SS, squalene synthase; SE, squalene epoxidase, OSC, 2,3-oxidosqualene cyclase. (**F**) Heat map of annotated transcripts in triterpene biosynthetic pathway expressed in shoots and roots. The color from red to blue indicates high to low expression levels.

The terpenoid biosynthesis is supplied from the cytosolic mevalonate (MVA) pathway and the plastidial 2-C-methyl-D-erythritol 4-phosphate (MEP) pathway to generate terpene precursor, isopentenyl diphosphate (IPP), and dimethylallyl diphosphate (DMAPP) (Figure 4A,C,E). The RLE values for unigenes in the MVA and MEP pathway are visualized by heat map images in Figure 4B,D. The Heat map for unigenes in the IPP pathway is shown in Figure 4F. It is known that HMG-CoA reductase (HMGR) and squalene epoxidase (SE) are the limiting enzymes in upstream of triterpene biosynthesis. One HMGR unigene (TRINITY_DN1756_c0_g2_i2), one unigene (TRINITY_DN633_c1_g1_i6) of SS, and one unigene (TRINITY_DN28775_c1_g1_i6) of SE showed high expression value in both shoot and root (Figure 4D,F).

### 2.4. Isolation of Triterpene Synthase in C. lanceolata

**Figure 5:**
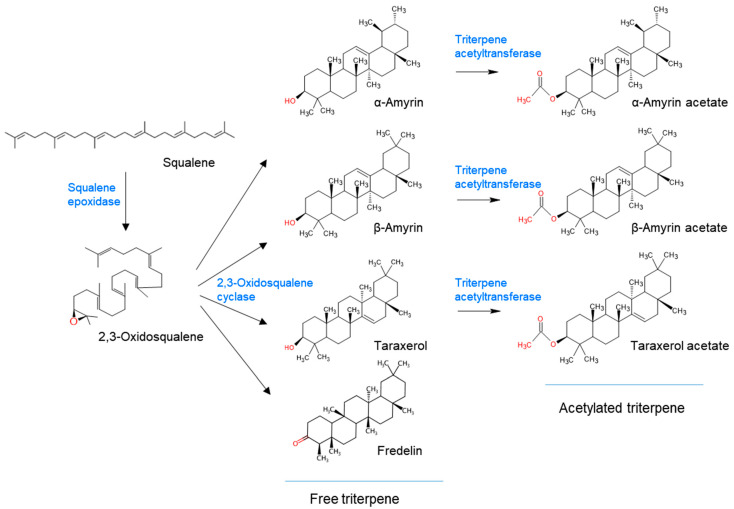
Biosynthetic pathway of triterpenes and triterpene acetates from squalene in *C. lanceolata* operates via squalene epoxidases, 2,3-oxidosqualene cyclases, and triterpene acetyltransferases.

Figure 5 shows that squalane is converted into squalene epoxide by squalene epoxidase, and then various triterpene skeletons are synthesized by triterpene synthase, oxidosqualene cylclase (OSC)., from squalene epoxides, and these substances are synthesized into triterpene acetates by triterpene acetyltransferase. In plants, various OSCs are participated to produce triterpene skeletons by the cyclization of 2,3-oxidosqualene [7]. These enzymes exist as supergene families in the plant genome. The genes involved in triterpenes and saponin biosynthesis in *C. lanceolata* have not been identified.

**Figure 6:**
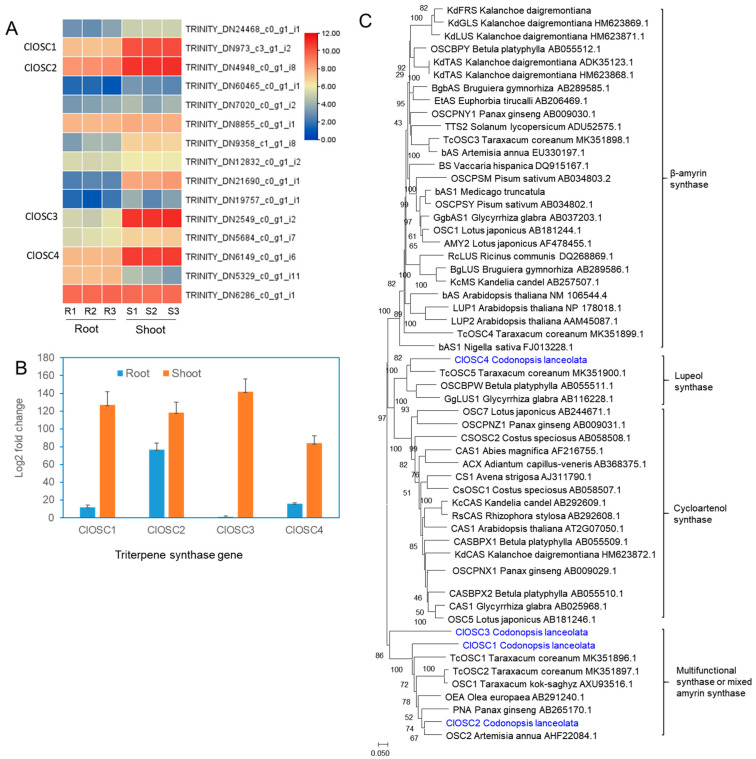
Transcriptional activity of 15 OSC unigenes, qPCR and phylogenetic analysis of selected four OSC genes of *C. lanceolata*. (**A**) Heat map analysis of the differential expression scale of annotated 15 OSC transcripts involved in triterpene production expressed in shoots and roots of *C. lanceolata.* (**B**) qPCR analysis for transcriptional activities of the four OSC mRNAs selected from 13 unigenes. The analysis results are presented as the mean ± SE of three independent experiments, each performed in triplicate. The expression of the actin gene was used as a normalization control. (**C**) Neighbour-joining phylogenetic analysis of the four OSCs (ClOSC1-4) isolated from *C. lanceolata* transcriptome and other characterized plant OSCs.

A total of 15 OSC sequences were obtained among annotated 39,523 unigenes (Figure 6A). Heat map analysis on the expression values of 13 OSC transcripts except for two transcripts (TRINITY_DN5329_c0_g1_i11 and TRINITY_DN6286_c0_g1_i1) showed higher expression in the shoots than the roots (Figure 6A). These four OSC transcripts were registered in GenBank, named as *ClOSC1* (accession number, ON186485), *ClOSC2* (accession number, ON186486), *ClOSC3* (accession number, ON186487), and *ClOSC4* (accession number, ON186488), respectively. Quantitative real-time PCR (qPCR) of selected four *OSC* sequences revealed that the relative expression levels of *ClOSC1*, *ClOSC2*, *ClOS3*, and *ClOSC4* were higher in the shoots than those in the root (Figure 6B). The RT-qPCR data showed the same expression trends of these gene transcripts in the transcriptome analysis. Phylogenetic analysis was conducted with deduced amino acid sequences of four ClOSCs (ClOSC1, ClOSC2, ClOS3, and ClOS4) together with those of other plant OSCs. Three *C. lanceolata* OSCs (*ClOSC1*, *ClOSC2*, *ClOS3*) were grouped to multifunctional triterpene synthase genes (Figure 6B). ClOSC4 is positioned in the lupeol synthase subgroup (Figure 6C). 

### 2.5. Functional Characterization of ClOSCs by Yeast Expression

**Figure 7:**
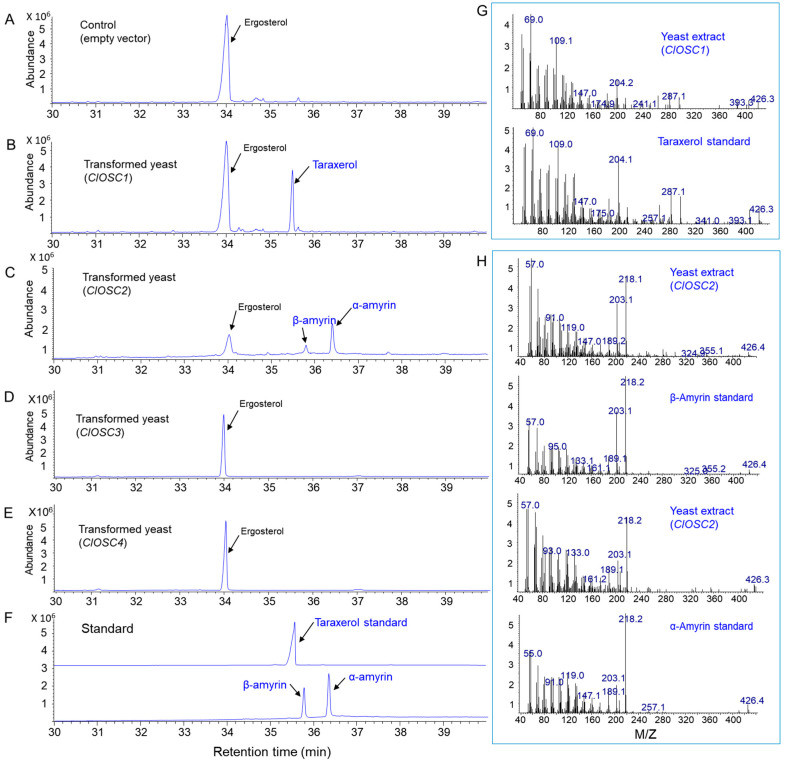
GC analysis of triterpenes extracted from yeast overexpressing the *C. lanceolata ClOSC1*, *ClOSC2*, *ClOSC3*, and *ClOSC4* genes. (**A**) Chromatogram of control yeast transformed with the empty vector. (**B**) Chromatogram of single triterpene product (taraxerol) in yeast transformed with the *ClOSC1*. (**C**) Chromatogram of two triterpene products (α-amyrin and β-amyrin) in yeast transformed with the *ClOSC2*. (**D**) Chromatogram of yeast extracts transformed with the *ClOSC3*. (**E**) Chromatogram of yeast extracts transformed with the *ClOSC4*. (**F**) GC chromatogram of the authentic taraxerol standard and α-amyrin and β-amyrin standards. (**G**) MS spectra for the taraxerol peak in the extracts of transgenic yeast expressing *ClOSC1* compared to those of the taraxerol standard. (**H**) MS spectra for the β-amyrin peak and α-amyrin peaks in the extracts of transgenic yeast expressing *ClOSC2* compared to those of the β-amyrin and α-amyrin standards.

To functionally analyze these four OSC genes, each ORF sequence was expressed in yeast. GC/MS analysis revealed that the total ion chromatogram (TIC) of extracts from transgenic yeasts expressing the empty vector showed only chromatogram peaks of the intrinsic compound, such as ergosterol (Figure 7A). The TIC for the extracts of transgenic yeast expressing *ClOSC1* revealed one new triterpene product at a retention time of 35.7 min (Figure 7B), which matched the retention time of the taraxerol standard (Figure 7F). The mass fragmentation patterns of the taraxerol peak in the transgenic yeast showed the same patterns as the authentic taraxerol standard (Figure 7G). The TIC for the extracts of transgenic yeast expressing *ClOSC2* showed the production of two new triterpenes (β-amyrin and α-amyrin) (Figure 7C). These two peaks detected in transgenic yeast expressing *ClOSC2* are matched with the β-amyrin and α-amyrin standards (Figure 7F). The mass fragmentation patterns of the two peaks in the TIC were the same as those of the authentic β-amyrin and α-amyrin standards (Figure 7H). Extracts from transgenic yeast expressing *ClOSC3* and *ClOSC4* revealed no functional product of triterpenes (Figure 7D,E).

### 2.6. Isolation of Putative Triterpene Acetyltransferase Gene Involved in the Production of Triterpene Acetates

**Figure 8:**
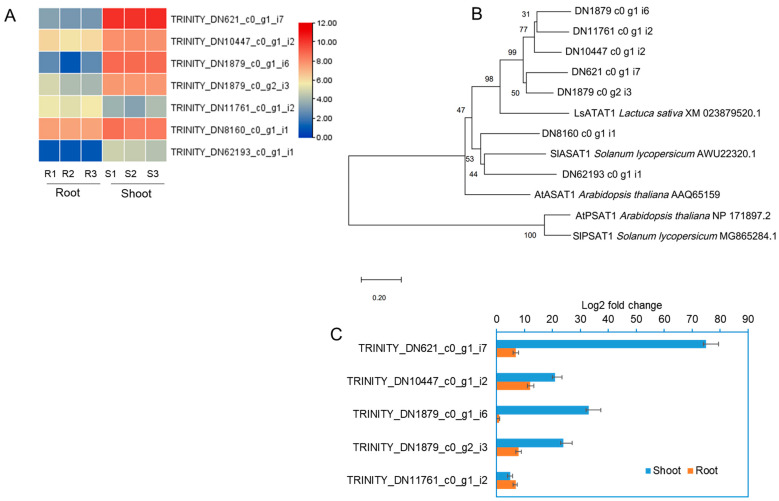
Transcriptional activity and phylogenetic analysis of putative triterpene acetyltransferase and sterol acetyltransferase unigenes of *C. lanceolata*. (**A**) Heat map analysis of the differential expression scale of annotated 7 sterol acetyltransferase unigenes expressed in shoots and roots of *C. lanceolata*. (**B**) Neighbour-joining phylogenetic analysis of the seven sterol acetyltransferase-like unigenes isolated from *C. lanceolata* transcriptome and other characterized plant enzymes. (**C**) qPCR analysis for transcriptional activities of the five unigenes having homology to Lettuce LsTAT1. The analysis results are presented as the mean ± SE of three independent experiments, each performed in triplicate. The expression of the actin gene was used as a normalization control.

GC analysis revealed that various types of triterpene acetates present in *C. lanceolata* plants, and shoots of *C. lanceolata* contained richer amount of triterpene acetates than free-triterpenes (Figure 1). It has been reported that triterpene acetate is converted from triterpenes by triterpene acetyltransferase [14]. Plant triterpene acetyltransferase gene was recently identified for the first time in lettuce, and the triterpene acetyltransferase (LsTAT1) sequence of lettuce is similar to acyl-CoA:sterol acyltransferases, but converts taraxasterol, a pentacyclic triterpene, to taraxasterol acetate without acetylation of sterol [14]. Seven contigs were found annotated as acyl-CoA:sterol acyltransferases in the transcripts of *C. lanceolata* transcriptome. The heat map reveals that the expression level of six contigs was high in the stem except for one contig (TRINITY_DN1761_c0_g1_i2) (Figure 8A). 

Phylogenetic analysis showed that 5 out of 7 contigs of *C. lanceolata* belong to the same group as lettuce triterpene acetyltransferase (LsTAT1), and the other remaining 2 contigs belong to the same group as the acyl-CoA:CoA: sterol acyltransferases in *Arabidopsis* and *Solanum lycopersicum* (Figure 8B). *Arabidopsis* phospholipid: sterol acyltransferase (AtPSAT1) and *S. lycopersicum* phospholipid: sterol acyltransferase (SlPSAT1) were positioned in the independent group (Figure 8B). qPCR of 5 unigenes having high homology to the LsTAT1 revealed that the relative expression levels of TRINITY_DN621_c0_g1_i7 showed the highest expression in shoots compared to the other 4 unigenes (Figure 8C).

## 3. Discussion

We identified the four triterpenoids and three triterpene esters by GC-MS in the roots and shoots of *C. lanceolata* plants. Triterpene acetates were richer in aerial parts of *C. lanceolata* plants compared to free triterpenes except for taraxerol. Although triterpene occurrence was reported in some articles such as friedelin [23] and taraxerol [23] in *C. lanceolata*, our data is the first report on the simultaneous identification of various triterpene and triterpene esters including α-amyrin, β-amyrin, α-amyrin acetate, β-amyrin acetate, and taraxerol acetates. It has known that *C. lanceolata* plants also contain several saponins (triterpene glycosides) [6]. The main glycosylated triterpenes in *C. lanceolata* are lancemaside A and B, foetidissimoside A, and aster saponin, which are known to be saponins derived from β-amyrin [6]. Therefore, it is considered that the presence of β-amyrin and β-amyrin acetate in the stems and roots of *C. lanceolata* is not special. The above results indicate that *C. lanceolata* plants contain various types of free triterpenes, triterpene esters, and triterpene glycosides. The contents of free triterpene, triterpene esters, and triterpene glycosides may be controlled in a tissue-specific and developmentally regulated manner.

Next-generation sequencing technology (NGS) is an effective approach for gene discovery involved in important secondary metabolites in medicinal plants. Transcriptome analysis has been widely used to discover the genes involved in the biosynthetic pathways and regulatory mechanisms of key metabolites related to medicinal compounds in many medicinal plants [26]. No transcriptomic sequencing data is available in *C. lanceolata* except for a relative species (*C. pilosula*) for the identification of genes involved in polysaccharide biosynthesis [17]. Thus, our article is the first transcriptome analysis using *C. lanceolata*. This study would be helpful to understand the role of genes involved in triterpenoid biosynthesis in *C. lanceolata.*

De novo sequencing of mRNAs isolated from shoots and roots of *C. lanceolata* plants was performed to discover the genes involved in triterpene biosynthesis. Terpenoid backbones are built up from C5 units, IPP and DMAPP. These two isoprenes are supplied from the cytosolic MVA and MEP pathways. Triterpenoid precursors are mainly supplied from the MVA pathway but also partly supplied from the MEP pathway [10,15]. We investigated the transcriptional activity of MVA and MEP pathway genes in the upstream region of triterpene biosynthesis. The transcriptional activity of unigenes was higher in the MVA pathway than in the MEP pathway. This is might be due to the active biosynthesis of triterpenoids in *C. lanceolata.* Moreover, transcription values of genes in the upstream pathway of triterpene biosynthesis were generally higher in shoots than those in roots. This result corresponds with the higher content of triterpenes and triterpene acetates in the shoots than in the roots of *C. lanceolata*.

The first step in triterpene biosynthesis is the production of squalene by condensation of two molecules of FPP by squalene synthase. This molecule is then activated by epoxidation by a squalene epoxidase, resulting in 2,3-oxidosqualene. In *C. lanceolata* transcriptome, four unigenes of squalene synthase and five unigenes of squalene epoxidase were found. *Platycodon grandiflorum* is related to *Codonopsis* species belonging to the same family Campanulaceae. One unigene of squalene synthase and 10 unigenes of squalene epoxidase were detected in the annotated transcriptome of *P. grandiflorum* [1]. Generally, squalene epoxidase has multiple enzymes and has a functional role in the rate-limiting enzyme that regulates either sterol or triterpenoid biosynthesis [27,28,29]. Suzuki et al. [25] suggest that different isoforms of squalene epoxidase genes play different roles with respect to triterpene and sterol biosynthesis. Six squalene epoxidase isoforms (*SQE1-6*) were identified in *Arabidopsis thaliana* [28]. Rasbery et al. [28] found that the *Arabidopsis* SQE1, SQE2, and SQE3 were functional squalene epoxidase. In contrast, SQE4, SQE5, and SQE6 were not functional. They suggested that squalene epoxidase genes have different isoform-dependent functions in *Arabidopsis*. In *Panax ginseng*, expression of two squalene epoxidase genes, *PgSQE1* and *PgSQE2*, were regulated in a different manner, and *PgSQE1* regulates ginsenoside biosynthesis, but not that of phytosterols [29]. Thus, the several isoforms of squalene epoxidase in C. *lanceolata* may reveal the different functional activities among squalene epoxidase isoforms for the biosynthesis of sterols and triterpenes.

The first diversifying step in triterpenoid biosynthesis is the cyclization of 2,3-oxidosqualene catalyzed by OSCs [30,31]. We obtained 15 OSC unigenes among a total of 39,523 representative transcripts in *C. lanceolata.* Four transcripts (TRINITY_DN973_c3_g1_i2, TRINITY_DN4948_c0_g1_i8, TRINITY_DN2549_c0_g1_i2, and TRINITY_DN6149_c0_g1_i6) showed high read number compared to other transcripts. We selected the four unigenes for further analysis and named to *ClOSC1-4* genes. Phylogenetic analysis revealed that the amino acid sequences of the first three genes (*ClOSC1*, *ClOSC2*, and *ClOSC3*) are grouped into a cluster having multifunctional triterpene synthase and/or mixed amyrin synthase genes. ClOSC4 is positioned in the lupeol synthase subgroup. The four OSC genes were functionally analyzed by yeast expression. ClOSC1 is determined taraxerol synthase and ClOSC2 is mixed amyrin synthase producing two triterpenes (α-amyrin and β-amyrin). Two others (ClOSC3 and ClOSC4) showed no functional product when expressed in yeast. Thus, the two enzymes (ClOSC3 and ClOSC4) may be encoded by pseudogenes having little or no enzyme catalysis, although they showed high transcriptional activity. In other plants, when the function of the *OSC* gene is analyzed through yeast expression, pseudogenes with no function are frequently observed [32,33,34]. In rice, 3 out of 6 *OSC* genes were found to be pseudogenes [34]. In our study, the ClOSC4 is similar to the lupeol synthase gene, but was found to have no function. This result is consistent with the fact that lupeol was not observed even when the shoots and roots of *C. lanceolata* were analyzed. Because we found the four triterpenes (taraxerol, β-amyrin, α-amyrin, and friedelin) in *C. lanceolata* plants, other uncharacterized 9 *OSC* sequences may be still have novel functional activity for triterpene production including friedelin. 

Plant triterpene acetyltransferase gene is recently identified in lettuce [14]. The lettuce triterpene acetyltransferase (LsTAT1) is similar to *Arabidopsis* acyl-CoA:sterol acyltransferase (AtASAT1), which converts taraxasterol, a pentacyclic triterpene, to taraxasterol acetate [14]. Seven contigs were annotated as acyl-CoA:sterol acyltransferases in the transcripts of *C. lanceolata* transcriptome. Phylogenetic analysis showed that 5 out of 7 contigs of *C. lanceolata* belong to the group of lettuce triterpene acetyltransferase (LsTAT1), and the other remaining 2 contigs belong to the group of sterol acyltransferases in *Arabidopsis* and *S. lycopersicum*. Therefore, the five contigs belonging to the group of LsTAT1 are the best candidate genes involved in triterpene acetyltransferase in *C. lanceolata.* Particularly, TRINITY_DN621_c0_g1_i7 unigene showed the highest expression compared to the other four unigenes, indicating this unigene might be the best candidate gene of triterpene acetyltransferase in *C. lanceolata*.

## 4. Materials and Methods 

### 4.1. Plant Materials

Seeds of *C. lanceolata* were purchased from Asia Seed Co., Ltd. (Seoul, Korea). The experiments were performed by relevant national and international guidelines and regulations. Fresh shoots and roots of *C. lanceolata* (2 months old) germinated from seeds in plastic plug trays were collected to isolate mRNA and to analyze the triterpenes and triterpene esters. Three independent biological replicates (i.e., derived from three plants) of each organ were washed with water, frozen in liquid nitrogen, and then stored at −80 °C until RNA and metabolite extraction.

### 4.2. RNA Isolation and cDNA Library Construction 

Total RNAs were purified in triplicate from the shoots and roots of *C. lanceolata* plants using an RNeasy plant mini kit (Qiagen, Hilden, Germany) according to the manufacturer’s instructions. Quality and quantity of RNA were examined using the Agilent 2100 BioAnalyzer (Agilent technologies, Waldbronn, Germany), with an RNA integrity value greater than seven (Agilent Technologies, Santa Clara, CA, USA). A total of six libraries was constructed using the Illumina TruSeq RNA sample preparation kit (Illumina, CA, USA). Total mRNAs enriched by poly A tail selection. Chemically fragmented mRNAs using the Ambion RNA fragmentation kit (Ambion, Austin, TX, USA) were used for first-strand cDNA synthesis and followed by second-strand cDNA synthesis using random hexamers. The blunted cDNAs were synthesized by adding adenine nucleotides and connected with sequencing adaptors. After size selection of the target cDNA fragments in the libraries, polymerase chain reaction (PCR) was performed using adaptor primers. The sequencing libraries were quantified using the Kapa library quantification kit (Roche, Switzerland) according to the manufacturer’s instructions.

### 4.3. Illumina Sequencing and De Novo Assembly 

All cDNA libraries were sequenced using the HiSeq X Ten System (*Illumina*, San Diego, CA, USA). From the sequenced transcriptome short reads, the adapter sequence was removed with Cutadapt [35] and SolexaQA software (v.2.4) [36] to exclude bad quality bases present at both ends of the short reads and reads of 25 bp or less.

De novo assembly of the trimmed reads was performed using the Velvet (v1.2.10) [37] and Trinity assembler tool [38]. Raw sequencing data of RNA-Seq were deposited in the National Center for Biotechnology Information (NCBI) sequencing read archive under accession number, PRJNA827661 (https://dataview.ncbi.nlm.nih.gov/object/PRJNA827661 (accessed on 18 April 2022)).

Trimmed reads for each sequence tag were mapped to the assembled transcripts using Bowtie software, accessed on 25 January 2022 [39]. The number of mapped clean reads for each unique transcript was calculated using an in-house script. Gene expression datasets were generated from each of two differential stages and raw read counts were normalized using the Relative Log Expression (RLE) normalization method implemented in the DESeq package in the R software (version“4.0”) [40]. The fold change and the number of reads mapped to each unigene were used to identify DEGs between the two sequencing samples. A false discovery rate was applied to calculate the *p*-values for statistical significance in multiple-comparison tests. 

### 4.4. Functional Annotation and DEG Selection

All assembled transcripts from the total RNA-seq reads were validated by direct comparison using the non-redundant (NR) databases (https://www.ncbi.nlm.nih.gov/refseq) and UniProtKB (http://www.ebi.ac.uk/uniprot) databases and the euKaryotic Orthologous Groups (KOG) tool (http://www.ncbi.nlm.nih.gov/KOG) using BLASTx (e-value ≤ 1 × 10^−10^). DEG selection is a 2-fold change method that identifies a difference in expression of more than 2-fold in samples where the expression value mapped to each gene is compared with each other, and a method that satisfies the adjust *p*-value (FDR) of 0.01 or less. If the value of log_2 (Fold Change) is greater than 1, it is called up-regulation, and if it is less than −1, it is called downregulation. Gene ontology (GO) analysis was carried out using the sequence similarities (e-value ≤ 1 × 10^−10^) of proteins [41] and classified into functional categories such as BP (Biological Process), CC (Cellular Component), and MF (Molecular Function).

### 4.5. qPCR Analysis

The expression of four OSC genes (*ClOSC1*, *ClOSC2*, *ClOSC3*, and *ClOSC4*) and five unigenes of *C. lanceolata* was analyzed by qPCR. The expression of the actin gene was used as a normalization control. Primer sequences for four OSC genes are listed in Table S1. Primer sequences for five unigenes of *C. lanceolata* are listed in Table S2. Total RNAs from the root and shoot of *C. lanceolata* were reverse-transcribed into the first-strand cDNA using PrimeScript II 1st Strand cDNA Synthesis Kit (Takara, Japan). RT-qPCR analysis of gene expression was performed using three biological replicates and three technical replicates, with TB Green^®^ Premix Ex Taq™ II (Takara) and Agilent StrataGene Mx3000P qPCR System (Agilent, USA). The relative gene expression was analyzed using the 2^−ΔΔCT^ method.

### 4.6. Phylogenetic Analysis of Amino Acid Sequences of Putative Triterpene Synthase and Triterpene Acetyltransferases

The phylogenetic tree was constructed using the neighbor-joining method with MEGA 6.0 software (www.megasoftware.net, accessed on 25 January 2022) with bootstrapping with 1000 replicates to estimate the strength of the nodes in the phylogenetic tree [42].

### 4.7. Characterization of the four OSC Enzymes 

RNAs were extracted from the leaves of *C. lanceolata* using an RNeasy plant mini kit (Qiagen, Hilden, Germany) and reverse transcribed to generate cDNA. The primers for cloning *ClOSC1-4* genes are shown in Table S3. The PCR products of the four genes were ligated into a pYES2.1/V5-HIS-TOPO vector (Invitrogen, Waltham, MA, USA) and transformed into *Escherichia coli.* After gene sequences of plasmids, plasmids were transformed into the erg7 yeast mutant (MATa erg7 ura3-1 trp1-1) via electroporation. The culture of transformed yeasts and extraction of triterpenes followed the protocols described previously [14].

### 4.8. GC/MS Analysis

Shoots and roots of two-month-old *C. lanceolata* plants were air-dried at 50 °C in a drying oven. The milled powders (200 mg) from each sample were soaked in 100% methanol (1 mL) and sonicated for 30 min at a constant frequency of 20 kHz at 40 °C. The supernatant after centrifugation was filtered through a syringe filter. 

To analyze triterpene production in transgenic yeasts expressing the *ClOSC1-4* genes, the yeast cells were harvested by centrifugation (3000× *g* for 5 min), mixed with 80% methanol, and sonicated for 30 min. After centrifugation, the supernatant was transferred to a new tube containing 100% chloroform and then vortexed. The chloroform layer was obtained and subsequently filtered through a syringe filter.

An aliquot (1 µL) was taken for analysis using a gas chromatograph (Agilent 7890A) linked to an inert MSD system (Agilent 5975C) with the triple-axis detector and equipped with a HP-5MS capillary column (30 m × 0.25 mm, film thickness 0.25 mm). The inlet temperature was 250 °C and the column temperature was programmed to start at 150 °C for 5 min, increase to 300 °C at the rate of 5 °C/min, and hold for 20 min. The carrier gas was He with a flow rate of 1.2 mL/min. The ionization chamber temperature was 250 °C with 70 eV ionizing energy.

Chromatogram peaks in the GC analysis were identified by comparing with retention times of their authentic standards and mass fragmentation spectra. The α-amyrin, β-amyrin, taraxerol, and friedelin were used as standards for GC/MS analysis (Sigma-Aldrich Inc., Saint Louis, MO, USA). Moreover, α-amyrin acetate, β-amyrin acetate, and taraxerol acetate were purchased from ChemFaces Biochemical Co. Ltd. (Wuhan, China).

## 5. Conclusions

In this study, we identified the production of several triterpenes (*taraxerol,* α-amyrin, β-amyrin, and friedelin) in roots and/or shoots of *C. lanceolata*. The transcriptional activity of genes that participated in the triterpene biosynthetic pathway was investigated by *C. lanceolata* transcriptome analysis. After selection of the putative triterpene synthase genes (OSCs) involved in triterpene biosynthesis in *C. lanceolata*, two OSC enzymes, ClOSC1 and ClOSC2, functionally characterized as taraxerol synthse and mixed amyrin synthase producting both α-amyrin and β-amyrin. Additionally, we selected the putative triterpene acetyltransferase involved in triterpene acetate production in *C. lanceolata.* The current study is a valuable basis for future research on enzyme characterization of putative triterpene synthase and triterpene acetyltransferase and metabolic engineering for *C. lanceolata* triterpene biotechnology.

## Data Availability

The transcriptome datasets generated in this study were deposited in the NCBI SRA (Sequence Read Archive) for the Bio project: PRJNA827661 (https://dataview.ncbi.nlm.nih.gov/object/PRJNA827661 (accessed on 18 April 2022)).

## Supplementary Materials

The following supporting information can be downloaded at: https://www.mdpi.com/article/10.3390/ijms24065769/s1.
