# Supplementary material for: De Novo Transcriptome Sequencing of Codonopsis lanceolata for Identification of Triterpene Synthase and Triterpene Acetyltransferase
Source: Int J Mol Sci. 2023 Mar 17;24(6):5769. doi: 10.3390/ijms24065769 (PMC10056628; doi:10.3390/ijms24065769)
Supplement: Supplementary file 1 [file ijms-24-05769-s001.zip › Table S1 qPCR primers of OSC sequences.pdf]

Table S1. qPCR primer sequence of OSC sequences

Gene name (GenBank accession number)	Primer	Sequences (5'-3')	Product size (bp)	Tm
ClOSC1 (ON186485)	Forward<br>Reverse	CCGGAAACTCATGCCCTCT<br>TCCGTAGAAGTTCCTCCATCCC	80	60
ClOSC2 (ON186486)	Forward<br>Reverse	CCGGACTAATCGTAGACAAGGTG<br>TGCTCAGAAGATCTATTCCGTTTTC	92	60
ClOSC3 (ON186487)	Forward<br>Reverse	CATGTCAAGCCTTGCAGTGA<br>GGGTATTTTCAGATCAATTCCGC	75	60
ClOSC4 (ON186488)	Forward<br>Reverse	CCGCTACGACGTTAAGCC<br>TCATCTTCAATCTTCACTTGTGCTA	102	60
Actin	Forward<br>Reverse	TTGACTGAGCGTGGTTATTCTTTC<br>TCTTCGCAGTTTCTAATTCCTGTTC	118	60
