# Supplementary material for: De Novo Transcriptome Sequencing of Codonopsis lanceolata for Identification of Triterpene Synthase and Triterpene Acetyltransferase
Source: Int J Mol Sci. 2023 Mar 17;24(6):5769. doi: 10.3390/ijms24065769 (PMC10056628; doi:10.3390/ijms24065769)
Supplement: Supplementary file 1 [file ijms-24-05769-s001.zip › Table S2 qPCR primers of putative triterpene acetyltransferase sequences.pdf]

Table S2. qPCR primer sequence of putative triterpene acetyltransferase gene sequences

Unigene name	Primer	Sequences (5’-3’)	Product size (bp)	Tm
TRINITY_DN621_c0_g1_i7	Forward	GCGGTGATGGTGGATATTG	101	60
	Reverse	AACACAAATCCTAAAGAATATGAC		
TRINITY_DN10447_c0_g1_i2	Forward	GTGTCTGGATATTGAAATTGTTATC	102	60
	Reverse	ACCCAGCACAAATCCCAATG		
TRINITY_DN1879_c0_g2_i3	Forward	TGAGTGTGGGTATTGAAATTG	107	60
	Reverse	ACTCGTACCCAGCAGAAAT		
TRINITY_DN1879_c0_g1_i6	Forward	GCTGTGGATATTGAAATCATAA	101	60
	Reverse	GAACCCATGACAAATCCCA		
TRINITY_DN11761_c0_g1_i2	Forward	GGCTGTTGTTATTGAAGGTGT	102	60
	Reverse	GAAGCAAACACAAATACCCAT		
