# Supplementary material for: De Novo Transcriptome Sequencing of Codonopsis lanceolata for Identification of Triterpene Synthase and Triterpene Acetyltransferase
Source: Int J Mol Sci. 2023 Mar 17;24(6):5769. doi: 10.3390/ijms24065769 (PMC10056628; doi:10.3390/ijms24065769)
Supplement: Supplementary file 1 [file ijms-24-05769-s001.zip › Table S3 ORF primers for cloning of OSC genes.pdf]

Table S3. PCR primer sequences for obtaining of open reading frame of four OSC genes

Gene name (GenBank accession number)	Primer	Sequences (5'-3')	Product size (bp)
ClOSC1 (ON186485)	Forward	ATGTGGAAGCTGAAGATAGC	2268
	Reverse	TTACTGAAGCCAAACTTGT	
ClOSC2 (ON186486)	Forward	ATGTGGAAGCTAAAGATAGCC	2289
	Reverse	TTACAACTTTTGGCTCAGCA	
ClOSC3 (ON186487)	Forward	ATGTGGAAGCTTAAGGTAG	2271
	Reverse	GTTCCAAGGCTATTGAACA	
ClOSC4 (ON186488)	Forward	ATGTGGAAGCTGAGGACT	2277
	Reverse	TTATGCATGTAGAACATGACG	
